# Insights into insulin analog cross-reactivity: a comparative study of Siemens Atellica and LC-MS/MS

**DOI:** 10.1007/s12020-024-03970-6

**Published:** 2024-07-19

**Authors:** Jieli Li, Maya Hatten-Beck, Jason K. Y. Lee, Andrew N. Hoofnagle

**Affiliations:** 1 Department of Pathology, The Ohio State University Wexner Medical Center, Columbus, OH USA; 2 Department of Laboratory Medicine & Pathology, University of Washington, Seattle, WA USA; 3 Department of Medicine, University of Washington, Seattle, WA USA; 4 Department of Clinical Laboratory, University Hospital, The Ohio State University Wexner Medical Center, Columbus, OH USA

**Keywords:** Insulin, Insulin analog, Siemens Atellica, LC-MS/MS, Cross-reactivity

## Abstract

**Background:**

To address the challenges posed by inconsistent detection of analog insulin in commercially available insulin immunoassays, resulting in potential discrepancies in clinical findings and misdiagnosis during the investigation of factitious hypoglycemia., we aimed to evaluate the ability of the Siemens Atellica automated immunoassay to detect insulin analogs compared with LC-MS/MS.

**Methods:**

Five insulin analogs were analyzed at 10 ng/mL spiked into serum samples, with recombinant human insulin as positive controls. Insulin and C-peptide assays were performed using Siemens Atellica and LC-MS/MS. Recovery rates were calculated.

**Results:**

Siemens Atellica immunoassay demonstrated robust cross-reactivity (92–121%) of insulin analogs. In contrast, glargine was detected by LC-MS/MS but other analogs were not observed (<10% recovery).

**Conclusion:**

Our results indicate that the insulin assay conducted on the Siemens Atellica platform could be used to diagnose factitious hypoglycemia by detecting the specific insulin analogs involved. The findings from our studies indicate the suitability of this method for clinical laboratory use in cases where factitious hypoglycemia is under consideration as a potential diagnosis. Clinicians should take these results into account when interpreting insulin measurements, particularly in instances where insulin analog overdose is suspected.

## Introduction

Insulin, a hormone synthesized in the β-cells of the islets of Langerhans, plays an important role in regulating glycemia. In diabetic patients, insulin supplementation is often required for life and is administered as recombinant human insulin or insulin analogs [1, 2]. Both insulin and C-peptide, which are secreted in equimolar amounts from the β-cells, are useful in assessing endogenous insulin secretion. Moreover, insulin and C-peptide levels are helpful for monitoring the success of pancreatic transplants in restoring endocrine function of pancreatic β-cells and to motivate a reduction of insulin therapy [3, 4].

These measurements also play a role in the workup and management of unexplained hypoglycemia. Hypoglycemia presents a significant health concern, resulting from various factors such as diabetes medications, alcohol consumption, and critical illnesses like sepsis, chronic malnutrition, or organ failure. Factitious hypoglycemia, resulting from covert use of insulin or insulin secretagogues, must be considered in hypoglycemia diagnoses. This consideration often affects the patient-clinician dynamic, fostering feelings of deception in the clinician and mistrust in the patient. Factitious hypoglycemia, associated with insulin secretagogues, has been reported in multiple clinical scenarios, including medication errors and cross-reactivity in insulin testing [5–8]. Given its potential implications, factitious hypoglycemia should be assessed in all patients being evaluated for hypoglycemic disorders, particularly when episodes occur sporadically. In cases where exogenous insulin is implicated, significantly elevated plasma insulin levels and suppressed C-peptide levels during hypoglycemia confirm the absence of endogenous insulin secretion. In most clinical settings, simultaneous measurement of plasma insulin and C-peptide can establish the diagnosis of factitious hypoglycemia [7, 9].

Insulin quantification assays are essential tools for evaluating insulin therapy compliance and detecting insulin overdose. They are also valuable in diagnosing hypoglycemia in non-diabetic individuals. However, the growing use of insulin analogs complicates diagnostic accuracy in cases of suspected factitious hypoglycemia. Detection limitations in conventional insulin immunoassays may wrongly suggest non-insulin-mediated hypoglycemia [10].

Despite being available for over four decades, no standardized method exists for measuring serum insulin [11]. Efforts to standardize insulin assays have yielded inconsistent results [12], prompting calls for harmonization through traceability to a reference standard which is liquid chromatography–tandem mass spectrometry [13]. Studies evaluating interference from insulin analogs in automated immunoassays have produced varied outcomes [5, 14, 15], emphasizing the need to assess commercial assays comprehensively for improved diagnostic accuracy in hypoglycemia management. In addition, there are no data describing the cross reactivity of insulin analogs in the Siemens Atellica assay. To address this gap, we aimed to undertake an evaluation of the cross-reactivity of rapid-acting (Lispro, Aspart) and long-acting (Detemir, Glargine) insulin analogs in the Siemens Atellica immunoassays. LC-MS/MS was used as a reference method, known for its excellent specificity.

## Methods

Five insulin analogs were spiked at 10 ng/mL in serum samples. Cross-reactivity was determined for the Siemens Atellica insulin immunoassay. Recombinant human insulin preparations were used as positive controls. The insulin analogs, derived from pen treatment units (100 U/mL or 4 × 10^7 ^ng/mL), were diluted in serum samples with very low insulin concentrations (10^−6 ^ng/mL) to achieve final concentrations of 10 ng/mL. Each spiked serum sample was aliquoted and frozen at −20 °C before analysis.

**Fig. 1:**
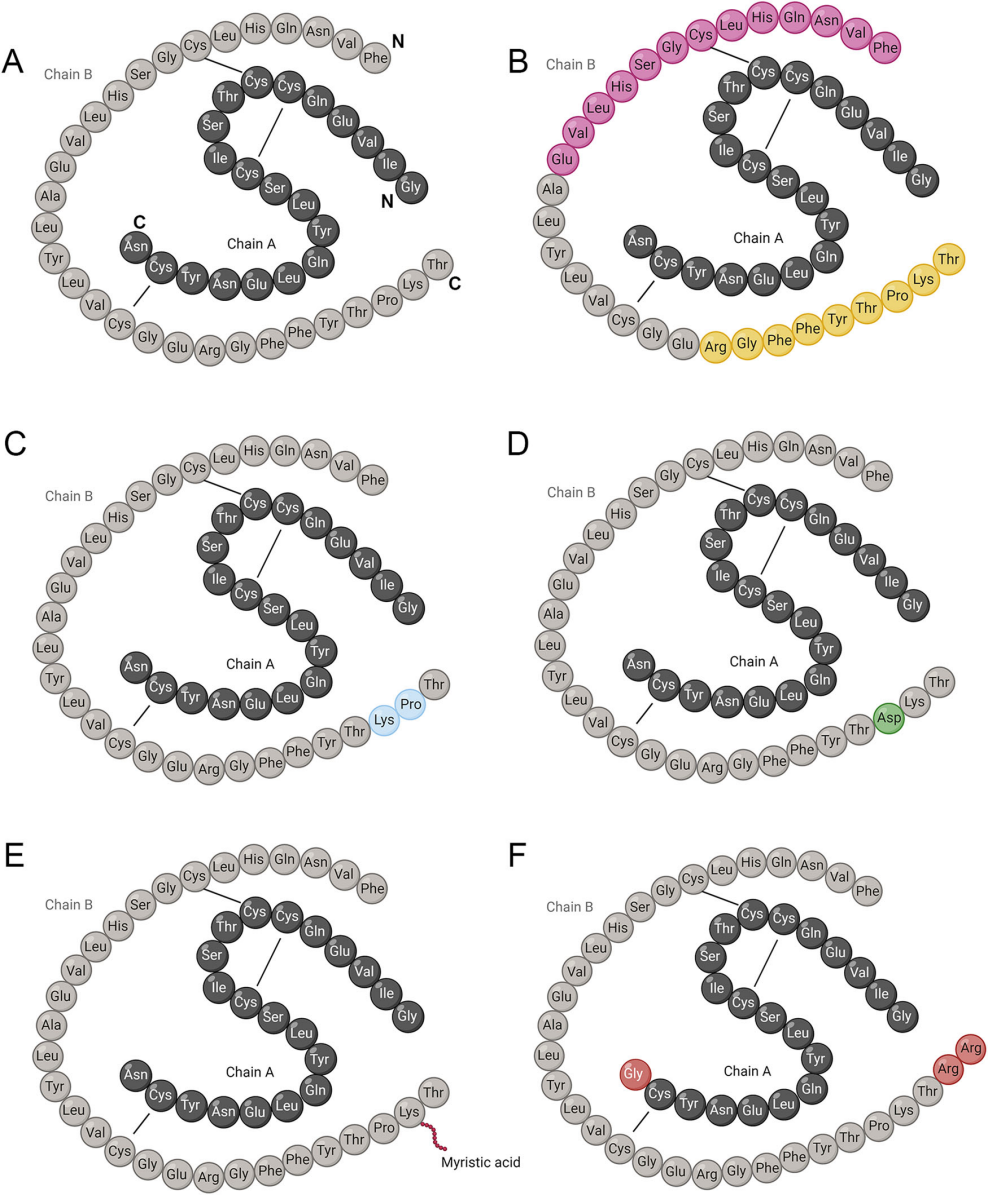
Structural Characteristics of Insulin Analogs and Detected Peptides on LC-MS/MS. **A** Human insulin structure. **B** Detected peptides of insulin by LC-MS/MS: RGFFYTPKT (proteotypic fragment used for quantitation) in yellow and FVNQHLCGSHLVE (for quality assurance) in pink. **C** HUMALOG® (Insulin Lispro) structure. **D** NovoLog® (Insulin Aspart) structure. **E** LEVEMIR® (Insulin Detemir) structure. **F** LANTUS (Insulin Glargine) structure

Structural differences of each insulin preparation are presented in Table 1 and Fig. 1 and provide a comprehensive overview of the insulin analogs under examination. Vendors of insulin analogs, including HUMALOG® (Lispro, 100 U/mL), NovoLog® (Aspart, 100 U/mL), LEVEMIR® (Detemir, 100 U/mL), LANTUS (Glargine, 100 U/mL), NovoLog® Mix 70/30 (Insulin aspart protamine and aspart), and human recombinant insulin [HUMULIN® R and HUMULIN® N (Isophane)], are listed in Table 1.

**Table 1:** Structural characteristics of commercial insulins

Name		Vendors	Structural characteristics	Figure panels
HUMALOG®	Insulin Lispro	Lilly, France	B28-29 Pro/Lys swapped	Fig. 1C
NovoLog®	Insulin Aspart	Novo Nordisk, Denmark	B28 Pro → Asp	Fig. 1D
LEVEMIR®	Insulin Detemir	Novo Nordisk, Denmark	B29 myristic acid	Fig. 1E
LANTUS	Insulin Glargine	Sanofi, France	A21 Asp →Gly, B31, 32, two additional Arg	Fig. 1F
NovoLog® Mix 70/30	Insulin Aspart protamine and insulin Aspart	Novo Nordisk, Denmark	70% aspart protamine and 30% aspart	
HUMULIN® R	Insulin human	Lilly, France	human sequence	
HUMULIN® N	Isophane insulin human suspension	Lilly, France	human sequence

Insulin and C-peptide assays were performed on a Siemens Atellica IM 1300 automated immunoassay analyzer and LC-MS/MS, as previously described [4]. The precisions of Insulin on Siemens Atellica, Level 1: mean 18.43 ng/mL, coefficient of variation (CV) 3.24%; Level 2: mean 74.07 ng/mL, CV 2.89%; Level 3: mean 172.74 ng/mL, CV 2.52%. The precisions of C-peptide on Siemens Atellica, Level 1: mean 1.23 ng/mL, CV 4.16%; Level 2: mean 5.05 ng/mL, CV 2.96%; Level 3: mean 14.97 ng/mL, CV 3.29%. For LC-MS/MS, two peptides from the insulin B-chain were monitored for native and isotopically-labeled internal standard insulin. The peptide RGFFYTPKT was used for quantitation (C-terminal, Fig. 1B yellow) and the peptide FVNQHLCGSHLVE was used for quality assurance (N-terminal, Fig. 1B pink).

The Atellica IM insulin assay employs a two-site monoclonal sandwich chemiluminescent immunoassay. Each sample (spiked and unspiked) was run in triplicate, and the mean was calculated. Percentage recoveries were determined by subtracting the mean of the unspiked sample from the spiked sample and dividing by 10 ng/mL. All units of insulin and C-peptide in the study were in ng/mL. Results were analyzed using Prism Graphpad Software, version 10.1.2.

## Results

**Fig. 2:**
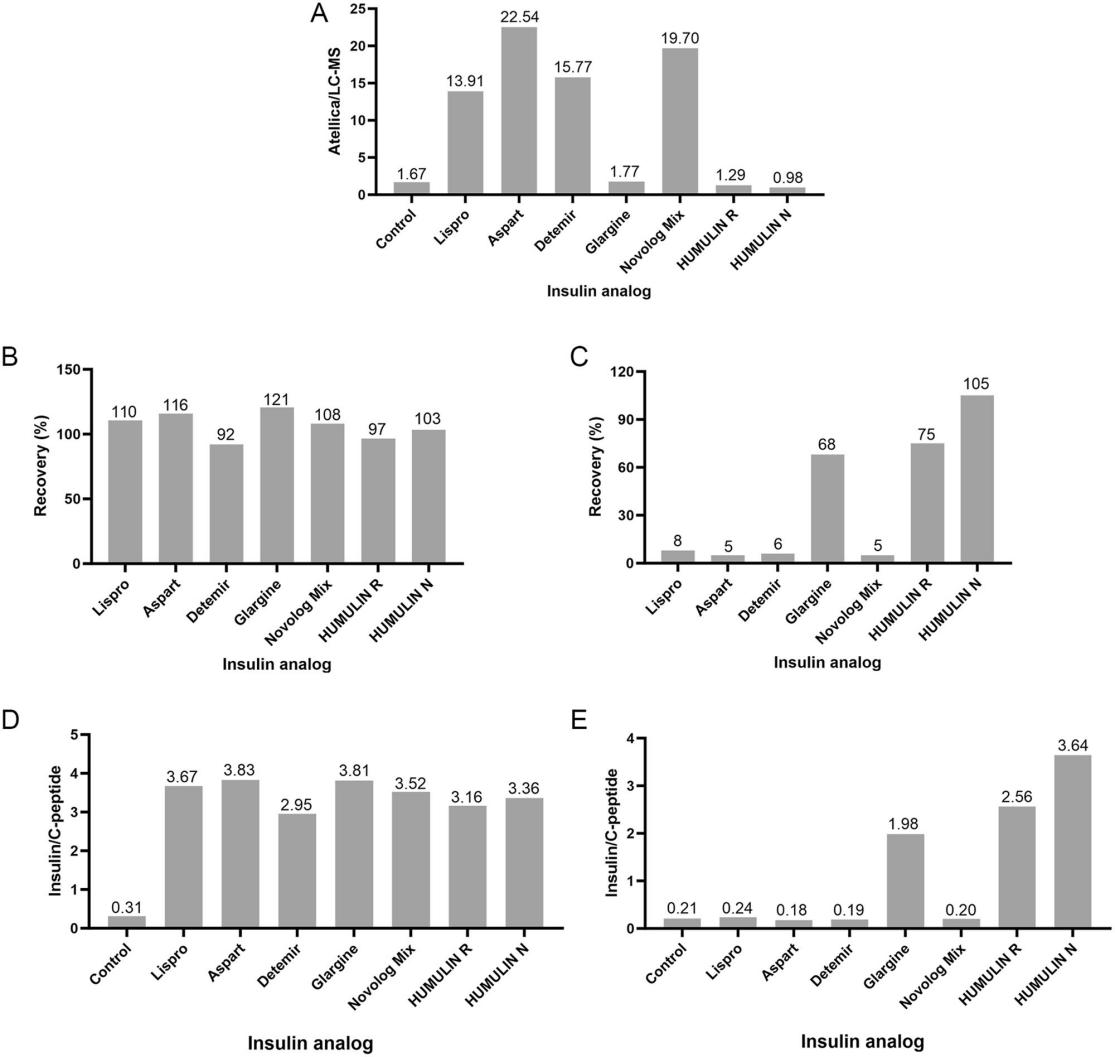
Insulin and C-peptide Measurements in Spiked Insulin Solutions by Siemens Atellica and LC-MS/MS. **A** Ratio of insulin results on Atellica to LC-MS/MS. **B** Insulin recovery rates on Atellica. **C** Insulin recovery rates on LC-MS/MS. **D** Ratio of insulin results (ng/mL) to C-peptide (ng/mL) on Atellica. **E** Ratio of insulin results (ng/mL) to C-peptide (ng/mL) on LC-MS/MS

The evaluation of the Siemens Atellica immunoassay and a LC-MS/MS assay focused on five insulin analogs added at a concentration of 10 ng/mL to serum samples to assess cross-reactivity. The Siemens Atellica immunoassay demonstrated robust detection, with recovery rates ranging from 92% to 121% for all insulin analogs (Fig. 2B), highlighting the potential of Siemens Atellica to accurately identify and quantify insulin analogs in serum samples. In contrast, LC-MS/MS, known for its specificity, did not detect most insulin analogs. However, glargine was an exception, demonstrating partial recovery (Fig. 2C).

When employing C-peptide as an internal reference, the difference in clinical interpretation between Siemens Atellica and LC-MS/MS was evident (Fig. 2D, E). The insulin to C-peptide ratios observed in spiked insulin lispro, aspart, detemir, and novolog mix samples on the Siemens Atellica platform were significantly higher compared to those obtained using LC-MS/MS. This finding aligns with the high recovery rates of insulin analogs observed on the Atellica platform, as illustrated in Fig. 2B, C. In contrast, the insulin to C-peptide ratio of glargine measured by LC-MS/MS was approximately 50% lower than that obtained on the Atellica platform. This discrepancy is consistent with the differences in recovery rates observed for glargine between LC-MS/MS and the Atellica platform. This highlights the significant difference of the detection of insulin analogs on Siemens Atellica and LC-MS/MS, indicating the different performance of Siemens Atellica with the gold standard LC-MS/MS.

## Discussion

Our study showed that the Siemens Atellica insulin immunoassay cross-reacted with all five insulin analogs (Lispro, Aspart, Aspart mix, Detemir, Glargine) with a recovery rate at 92–121%. The LC-MS/MS insulin assay did not cross-react with most insulin analogs except glargine, which is most likely due to metabolism of glargine ex vivo. These results shed light on the cross-reactivity of Siemens Atellica immunoassays with insulin analogs. The immunoassay proves effective in detecting various insulin analogs, offering clinicians a valuable tool in distinguishing between insulin of different origins. The distinct performance of LC-MS/MS, particularly in its minimal cross-reactivity, adds depth to our understanding of assay specificity.

Cross-reactivity is a function of the binding of the antibodies employed in the assay. Considering the structure of the studied analogs (Fig. 1), the results suggest that at least the Siemens Atellica insulin immunoassay is based on the use of antibodies recognizing the N-terminal part of the B-chain, which is not altered within the sequence of the analogs studied, hence the high cross-reactivity, while LC-MS/MS detected the C-terminal part of the B-chain which is altered within the sequence of the analogs studied [4]. These results confirm and extend the scope of cross reactivity reported in 2015 and 2023 using the other methods applied to these insulin analog measurements [14, 16].

Polyclonal antibodies used in other immunoassays provide less specificity compared to monoclonal antibodies. However, two monoclonal mice antibodies were used in the Atellica insulin assay, thereby reducing the specificity issue caused by cross-reactivity by polyclonal antibodies. Insulin detemir (Levemir®) comprises a modified parent insulin molecule bound to a fatty acid. In the previous study, the majority of commercial assays, with the exception of Mercodia Isoinsulin, BI-INS-IRMA and Abbott Architect showed no cross-reactivity with samples spiked with insulin detemir [14, 16], most likely due to the presence of large fatty acid groups preventing binding of anti-insulin antibodies. However, cross-reactivity still occurred on the Siemens Atellica, which when combined with the recovery rates from the other insulin analogs suggests that one of the binding sites near the C-terminus of the B-chain is at N-terminal to the proline.

Variable cross-reactivity of insulin analogs can lead to misdiagnosis with clearly established clinical impact. It is beneficial to know the capacity of individual assays to detect insulin analogs. When surreptitious insulin administration is suspected, different human insulin specific assays could be used in parallel with one insulin assay presenting significant cross-reactivity with insulin analogs (e.g., Siemens Atellica and LC-MS/MS). A case of artificial hypoglycemia by self-administration of lispro and glargine was noticed due to the discordant results from paired use of different immunoassays [17]. In another case report, despite a serum insulin concentration within the reference range, hypoglycemia was observed. The administrated glargine and insulin aspart escaped detection owing to low cross-reactivity between the analogs and human insulin in the insulin assays [5].

Mass spectrometry-based methods for insulin analog assessment have been described [4, 18–20]. However, these methods are technically demanding and not widely utilized. Human insulin immunoassays are easier to perform, but clinicians and clinical chemists must be aware that possible cross-reactivity has been highlighted between endogenous or exogenous standard insulin and analogs [21–23].

The limitation of the study is that this is an in vitro study only. A specific concern is insulin glargine, detected with the recovery rate at 121% on Siemens Atellica at the recovery rate, while nearly half (68%) on LC-MS/MS. Glargine is rapidly metabolized to its predominant active M1 metabolite in vivo, which is lacking the two carboxyl-terminal arginine residues [24]. When spiked glargine into serum, a significant signal for insulin was observed even with the LC-MS/MS method, confirming the rapid conversion of glargine in vitro due to active peptidases in serum. There could be M1 metabolite in vitro detected by Atellica. As a result of this interference, clinicians and clinical laboratories may still interpret the result with very low level of C-peptide in patients who are using glargine for glucose control, as the detectable insulin produced endogenously via either immunoassay or LC-MS/MS.

In summary, our study provides new information regarding the responsiveness of the Siemens Atellica immunoassay to insulin analogs. Clinical laboratories should understand the analytical specificity of their insulin immunoassay.
